# A Novel Fusion of Radiomics and Semantic Features: MRI-Based Machine Learning in Distinguishing Pituitary Cystic Adenomas from Rathke’s Cleft Cysts

**DOI:** 10.5334/jbsr.3470

**Published:** 2024-02-01

**Authors:** Ceylan Altintas Taslicay, Elmire Dervisoglu, Okan Ince, Ismail Mese, Cengizhan Taslicay, Busra Yaprak Bayrak, Burak Cabuk, Ihsan Anik, Savas Ceylan, Yonca Anik

**Affiliations:** 1 Department of Radiology, The University of Texas MD Anderson Cancer Center, Houston, TX, USA; 2 Department of Radiology, Kocaeli University, Kocaeli, Turkey; 3 Department of Vascular and Interventional Radiology, Rush University, Chicago, IL, USA; 4 Department of Radiology, Health Sciences University, Erenkoy Mental Health and Neurology Training and Research Hospital, Istanbul, Turkey; 5 Department of Mechanical Engineering, Rice University, Houston, TX, USA; 6 Department of Pathology, Kocaeli University, Kocaeli, Turkey; 7 Department of Neurosurgery, Pituitary Research Center, Kocaeli University, Kocaeli, Turkey; 8 Department of Neurosurgery, Pituitary Research Center, Kocaeli University, Kocaeli, Turkey; 9 Department of Neurosurgery, Pituitary Research Center, Kocaeli University, Kocaeli, Turkey; 10 Department of Radiology, Kocaeli University, Kocaeli, Turkey

**Keywords:** Cystic Pituitary Adenomas, Machine Learning Algorithms, Magnetic Resonance Imaging, Radiomics, Rathke’s Cleft Cysts

## Abstract

**Objectives:**

To evaluate the performances of machine learning using semantic and radiomic features from magnetic resonance imaging data to distinguish cystic pituitary adenomas (CPA) from Rathke’s cleft cysts (RCCs).

**Materials and Methods:**

The study involved 65 patients diagnosed with either CPA or RCCs. Multiple observers independently assessed the semantic features of the tumors on the magnetic resonance images. Radiomics features were extracted from T2-weighted, T1-weighted, and T1-contrast-enhanced images. Machine learning models, including Support Vector Machines (SVM), Logistic Regression (LR), and Light Gradient Boosting (LGB), were then trained and validated using semantic features only and a combination of semantic and radiomic features. Statistical analyses were carried out to compare the performance of these various models.

**Results:**

Machine learning models that combined semantic and radiomic features achieved higher levels of accuracy than models with semantic features only. Models with combined semantic and T2-weighted radiomics features achieved the highest test accuracies (93.8%, 92.3%, and 90.8% for LR, SVM, and LGB, respectively). The SVM model combined semantic features with T2-weighted radiomics features had statistically significantly better performance than semantic features only (*p* = 0.019).

**Conclusion:**

Our study demonstrates the significant potential of machine learning for differentiating CPA from RCCs.

## Introduction

Pituitary adenomas are the most frequent masses of the sellar region that typically show hypo- or isointense signals on magnetic resonance imaging (MRI) [1,2].When complicated with necrosis, hemorrhage, or cystic degeneration, their signals may change and even appear purely cystic [3].

Rathke’s cleft cysts (RCCs) originate from the embryologic remnants of Rathke’s pouch and often show similar signals to cerebrospinal fluid on T1-weighted imaging (T1WI) and T2-weighted imaging (T2WI). However, the diverse composition of the cystic fluid—ranging from serous to mucinous—may result in signal variability [4].

The differentiation between cystic pituitary adenomas (CPA) and RCCs is a significant challenge, as they often exhibit similar appearances on MRI. Existing research suggests that up to 50% of surgically confirmed RCCs were initially misidentified as CPA preoperatively [3].

The need for an accurate pre-surgical distinction between CPA and RCCs is of utmost importance as treatment strategies vary significantly. While RCCs may only require partial wall resection and cyst contents evacuation, CPA might necessitate total resection to mitigate mass effects and control hormonal excess [5-8]. Unnecessary surgical excision of RCCs can lead to severe complications, including cerebrospinal fluid leaks, infections, and hypothalamic injury [8,9]. Distinguishing CPA from RCCs based purely on MRI is a formidable task [10]. In light of this, developing reliable methods to improve preoperative differentiation is crucial. Our study delves into the potential of machine learning to meet this need.

## Materials and Methods

### Study Design and Data Collection

Approval for this study was obtained from the institutional review board under the Declaration of Helsinki. Contrast-enhanced pituitary MR images between 2015 and 2020 were reviewed retrospectively in our hospital’s Picture Archiving Communicating Systems workstation. A written informed consent form is provided to patients before routine MR examinations.

The inclusion criteria were as follows: (i) sellar location, (ii) a tumor size of >10 mm, (iii) pure cystic character, (iv) pathologically proven RCCs or CPA. The exclusion criteria were as follows: (i) mixed cystic-solid character, (ii) a tumor size of <10 mm, and (iii) low-quality MR images. A tumor size of 10 mm was taken as a cut-off value for inclusion criteria to evaluate semantic features more reliably, decrease interobserver variations, and segment the tumor more precisely. Finally, 65 patients (28 RCCs, 37 CPAs) were included in the study.

### MRI Acquisition

MR images were acquired at our center using 1.5 Tesla MRI (Gyroscan Intera, Philips Medical Systems, Eindhoven, The Netherlands) and 3 Tesla MRI (Achieva Intera, Philips Medical Systems, Eindhoven, The Netherlands) scanners equipped with a 16-channel head coil. Sagittal fat-saturated precontrast T1W images were acquired with a TE of 15 ms and a TR of 570 ms. Coronal T2W images were acquired with a TE of 120 ms and a TR of 3000 ms. Precontrast coronal and sagittal T1W images were acquired with a TE of 10 ms and a TR of 500 ms. All sequences were performed with a slice thickness of 3 mm, a gap of 0.3 mm, and a field of view (FOV) of 120 mm. Dynamic coronal T1W images were obtained with a TE of 10 ms, a TR of 500 ms, a slice thickness of 2 mm, a gap of 0.2 mm, and a FOV of 120 mm 3 minutes following the injection of the contrast.

### Semantic Feature Evaluation

**Figure 1:**
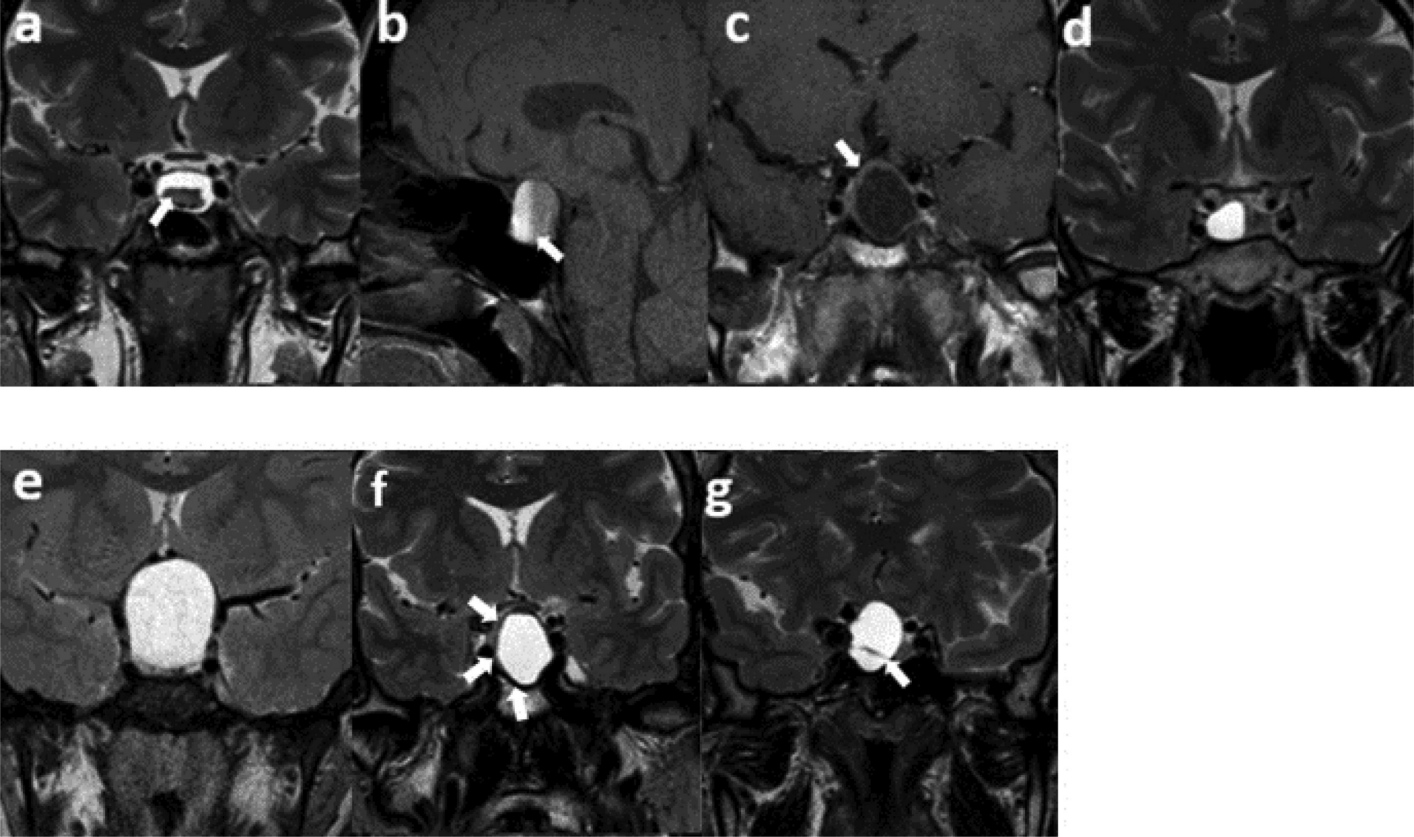
Semantic features. (a) An intracystic nodule on T2WI, (b) intralesional ﬂuid–ﬂuid level on SPIR T1WI, (c) ≥2 mm thickness of contrast-enhancing wall, (d) off-midline location, (e) suprasellar extension, (f) hypointense rim on T2WI, (g) intralesional septation on T2WI.

Two investigators blinded to the pathology results independently performed the initial semantic evaluation of the MRI. They assessed seven semantic features: intracystic nodules on T2WI, intralesional fluid-fluid levels on spectral presaturation with inversion recovery (SPIR) sagittal T1WI, contrast-enhancing walls with a minimum thickness of 2 mm on post-contrast T1WI, off-midline locations, suprasellar extensions, hypointense rims on T2WI, and intralesional septations on T2WI (Figure 1). These features were chosen due to their high reproducibility demonstrated in prior research [3,5,11]. An interobserver agreement analysis followed this initial assessment. If disagreements arose between the two observers, another senior observer with extensive experience in the field was consulted. The final decision was made by consensus.

### Radiomics Feature Extraction

**Figure 2:**
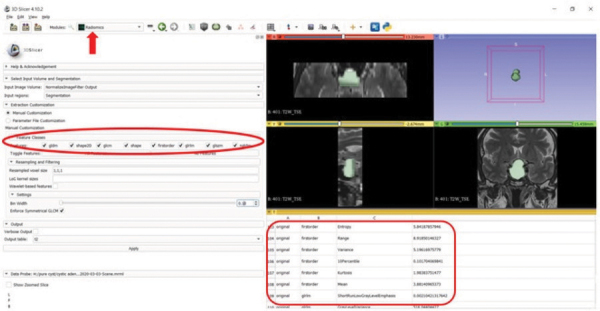
Segmentation and feature extraction (red rectangle, red ellipse) from the segmented volume using the Radiomics extension (arrow) of the 3D Slicer software on T2W image.

3D Slicer software version 4.10.2 (www.slicer.org) was used for tumor segmentation and radiomics analysis [12]. Following specific guidelines to ensure study reproducibility [13], the uploaded images underwent standardization using ± 3 sigma normalization and an N4ITK bias field correction filter. The voxel sizes were standardized to 1 × 1 × 1 mm³. Grey-level discretization was performed with a bin-width value of 0.1. All tumors were segmented independently by two observers (Figure 2). Fourteen shape-based features and 93 textural features, including 18 ﬁrst order, 24 Gray-Level Co-Occurrence Matrix (GLCM), 16 Gray-Level Run Length Matrix (GLRLM), 16 Gray-Level Size Zone Matrix (GLSZM), 14 Gray-Level Dependence Matrix (GLDM), and 5 Neighboring Gray Tone Difference Matrix (NGTDM) features, were extracted from SPIR T1WI, T2WI, and post-contrast T1WI, respectively, with the pyradiomics add-on feature [14].

### Feature Selection

In the 3-step feature selection process, the inter-observer reproducibility was first calculated for each radiomics feature using an intra-class coefficient (ICC) analysis [15]. Features with an ICC value of ≥0.75 were included in the further steps. Secondly, features having high collinearity in Pearson’s correlation analysis (a filter method) were excluded. The r threshold was selected as 0.7 [16]. Finally, the wrapper method was used as an additional layer for feature selection to find the optimal mix of features [17].

### Machine Learning Models

**Figure 3:**
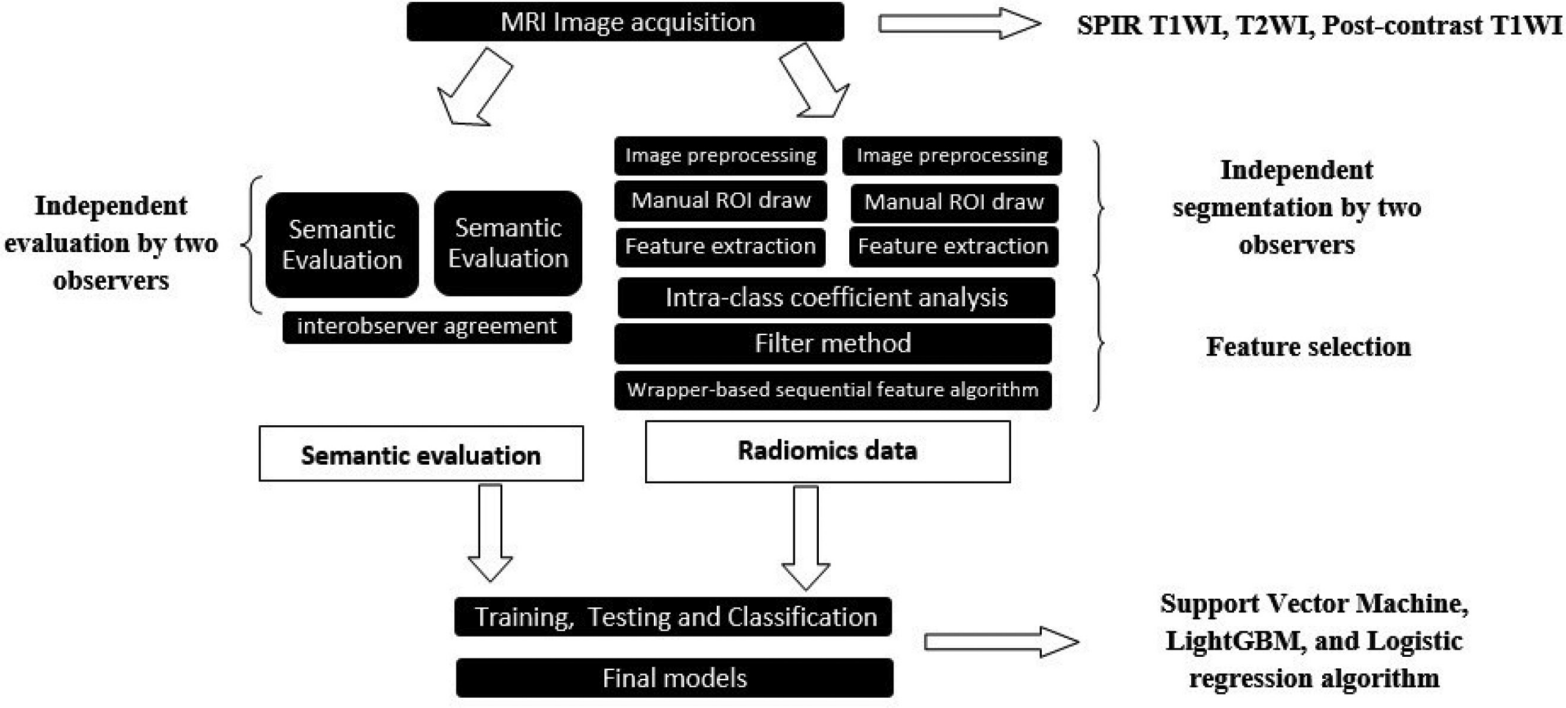
Schematic representation of the feature extraction process and the subsequent steps taken in developing machine learning models.

The selected features were subsequently incorporated into distinct machine-learning models. The Support Vector Machine (SVM), Light Gradient Boosting (LGB), and Logistic Regression (LR) were employed and executed in Python (version 3.7.11) for classification. Algorithms were carefully chosen based on their unique merits and alignment with the specific objectives of the research [18-20]. Five models were developed using five datasets as follows: semantic features only (semantic model), semantic features with T2WI radiomics features (T2W model), semantic features with T1WI radiomics features (T1W model), semantic features with postcontrast-T1WI radiomics features (T1C model), and semantic features with the combination of radiomics features obtained from all MRI sequences (the combined model). A nested 5-fold cross-validation (CV) method was assigned to all the models for evaluation. The hyperparameters of the models were optimized at the inner 5-fold CV loop, and performance metrics were evaluated at the outer 5-fold CV loop. Accuracy, sensitivity, specificity, F1 measure (a harmonic calculation of precision and recall), and area under the receiver operating characteristics curve (AUC) were calculated for each model. Figure 3 presents a schematic representation of the feature extraction process and the subsequent steps taken in developing machine learning models.

### Statistical Analysis

Statistical analysis was performed with the IBM SPSS 20.0 (SPSS Inc., Chicago, IL, USA) package program [21]. Comparisons between the groups for semantic features were made with the Chi-square test. Interobserver analysis of semantic features was performed by calculating Cohen-Kappa coefficients. Comparisons between the performances of the algorithms were analyzed with Friedman’s test. Statistically significant differences were further analyzed with post-hoc pairwise Wilcoxon tests. The statistical threshold was selected as p < 0.05.

## Results

The study population’s age range was 23 to 64 (44.1 ± 10) years. There were 22 females and 15 males in the CPA group, while the RCC group comprised 21 females and seven males. Upon conducting a demographic analysis, we found no significant difference in the distribution of gender between the CPA and the RCC groups (*p* = 0.295).

The interobserver agreement analysis of semantic features revealed kappa scores indicating moderate to high levels of agreement (Table 1).

**Table 1:** Interobserver agreement analysis for semantic features

SEMANTIC FEATURE	1ST OBSERVER	2ND OBSERVER		KAPPA AGREEMENT
		NONE	PRESENT	
**Fluid–Fluid level**	**None**	52	7	0.578 Moderate agreement
	**Present**	0	6	
**Septa**	**None**	37	5	0.428 Moderate agreement
	**Present**	11	12	
**Intracystic nodule**	**None**	44	10	0.491 Moderate agreement
	**Present**	2	9	
**Hypointense rim**	**None**	32	7	0.654 High agreement
	**Present**	4	22	
**Suprasellar placement**	**None**	14	3	0.490 Moderate agreement
	**Present**	12	36	
**Wall enhancement >2 mm**	**None**	40	9	0.512 Moderate agreement
	**Present**	4	12	
**Off-midline location**	**None**	16	16	0.442 Moderate agreement
	**Present**	2	31	

All semantic features except for suprasellar extension were significantly associated with the type of tumor (Table 2).

**Table 2:** Semantic features of CPA and RCCs

SEMANTIC FEATURE	CYSTIC PITUITARY ADENOMA *N* = 37	RATHKE CLEFT CYST *N* = 28	*P* VALUE
**Fluid–Fluid level**	6 (16.2%)	0 (0%)	0.003
**Septa**	19 (51.4%)	4 (14.3%)	< 0.001
**Intracystic nodule**	1 (2.7%)	18 (64.3)	< 0.001
**Hypointense rim**	20 (54.1%)	6 (21.4%)	0.008
**Suprasellar placement**	24 (64.9%)	24 (85.7%)	0.058
**Wall enhancement >2 mm**	16 (43.2%)	0 (0%)	< 0.001
**Off midline location**	24 (64.9%)	8 (28.6%)	0.008

Machine learning models that combined semantic and radiomic features achieved higher levels of accuracy than the models with semantic features only. The test AUC and train AUC were higher compared to the semantic models. T2W models achieved the highest test accuracies (93.8%, 92.3%, and 90.8% for LR, SVM, and LGB, respectively). Table 3 presents various metrics of different models on both the testing and training datasets, utilizing SVM, LR, and LGB algorithms.

**Table 3:** Various metrics of different models on both the testing and training datasets, utilizing SVM, LR, and LGB algorithms

DATASET	ALGORITHM	SEMANTIC MODEL	T2W MODEL	T1W MODEL	T1C MODEL	COMBINED MODEL
**Test accuracy**	**SVM** **LR** **LGB**	0.846 0.892 0.877	0.923 0.938 0.908	0.892 0.877 0.892	0.892 0.892 0.892	0.892 0.923 0.892
**Train accuracy**	**SVM** **LR** **LGB**	0.923 0.942 0.954	0.954 0.977 1.000	0.931 0.946 0.996	0.931 0.942 0.977	0.935 0.950 0.992
**Test AUC**	**SVM** **LR** **LGB**	0.956 0.956 0.951	0.960 0.980 0.945	0.956 0.970 0.980	0.980 0.981 0.954	0.990 0.985 0.961
**Test precision**	**SVM** **LR** **LGB**	0.905 0.898 0.975	0.925 0.928 0.933	0.902 0.880 0.921	0.888 0.909 0.913	0.884 0.927 0.928
**Test recall**	**SVM** **LR** **LGB**	0.836 0.943 0.807	0.950 0.975 0.918	0.918 0.918 0.889	0.943 0.914 0.914	0.943 0.943 0.889
**Test F1 score**	**SVM** **LR** **LGB**	0.853 0.913 0.876	0.937 0.950 0.922	0.906 0.894 0.900	0.911 0.904 0.904	0.909 0.933 0.901
**Test specificity**	**SVM** **LR** **LGB**	0.853 0.807 0.960	0.887 0.887 0.880	0.860 0.820 0.893	0.813 0.847 0.847	0.813 0.887 0.887

At the Friedman test, there was a significant difference in models with SVM and LR algorithms (*p* = 0.029 and *p* = 0.002, respectively). In the post-hoc pairwise analysis, the T2W model with SVM showed better performance than the semantic model with SVM (*p* = 0.019). On the other hand, both the T2W model and the combined model with the LR algorithm demonstrated better performance than the T1W model with LR (*p* = 0.007 and *p* = 0.035, respectively).

## Discussion

Our study underscores the remarkable capabilities of artificial intelligence in distinguishing CPA from RCCs based on MRI data. Leveraging a fusion of semantic features and radiomics, our models demonstrated significant accuracy in differentiating these entities.

Park et al.’s diagnostic tree model, derived from only imaging features, showed remarkable accuracy, with an AUC value of 0.991 [3]. Semantic models in our study with SVM, LR, and LGB showcased considerable accuracy with AUC values of 0.956, 0.956, and 0.951, respectively. Added radiomic features to semantic features in the combined models reinforced the robustness of the model’s predictive power. The AUC values of our combined models were quite promising (0.990, 0.985, 0.961, respectively).

Wang et al. employed a comprehensive semantic feature evaluation for distinguishing cystic adenomas from RCCs, detailing a variety of variables, including tumor shape, location, intensity on T1WI and T2WI, and intracapsular septation [10]. While their study achieved high diagnostic performance using the artificial neural network (ANN) classifier with semantic features (0.823 AUC), it is noteworthy that our study focused on more specific semantic features and identified significant correlations with tumor types using conventional statistical analysis. Also, we found better diagnostic performances with SVM, LR, and LGB classifiers using semantic features (AUC of 0.956, 0.956, and 0.951, respectively). Consistent with our current study, they reported that the combined radiomics and semantic model showed better diagnostic performance (0.848 AUC) than the semantic model. Our combined models with SVM, LR, and LGB classifiers showed AUC of 0.990, 0.985, and 0.961, respectively. In addition to their study, we also evaluated whether there was a significant difference between the models and found that the semantic and combined models showed no notable difference. On the other hand, the T2W model with SVM had statistically significantly better performance than the semantic model with SVM (*p* = 0.019). These variations emphasize the importance of model selection and highlight that certain algorithms may be more appropriate for specific tasks or datasets.

Although this research has demonstrated a moderate to high degree of consistency in the semantic features among observers, it is worth noting that there are disagreements among radiologists for some features. Transforming images into mineable data and allowing the analysis of a multitude of features, radiomics can offer higher consistency and may reduce human-related variability [13]. Their use could enhance diagnostic accuracy. Nevertheless, further research is required to optimize these processes and validate their utility across clinical settings and patient populations.

Despite the promising results, this study has some limitations. First, the sample size is relatively small, raising the potential for overfitting in the machine-learning models. Second, our study was retrospective and conducted at a single institution, which might limit the generalizability of the findings. Future research could include a larger sample size with multicenter prospective studies to validate these results.

## Conclusion

The high accuracy achieved by radiomics-driven machine learning models in our study demonstrates their potential utility as complementary diagnostic tools, refining the differential diagnosis between CPA and RCCs.
